# Improving the efficacy of osteosarcoma therapy: combining drugs that turn cancer cell ‘don't eat me’ signals off and ‘eat me’ signals on

**DOI:** 10.1002/1878-0261.12556

**Published:** 2019-08-13

**Authors:** Suchismita Mohanty, Maryam Aghighi, Ketan Yerneni, Johanna Lena Theruvath, Heike E. Daldrup‐Link

**Affiliations:** ^1^ Department of Radiology Molecular Imaging Program at Stanford Stanford University CA USA; ^2^ Department of Pediatrics Stanford University CA USA

**Keywords:** bone sarcoma, CD47, doxorubicin, ferumoxytol, macrophages, MR imaging

## Abstract

The long‐term survival of osteosarcoma patients with metastatic or recurrent disease remains dismal, and new therapeutic options are urgently needed. The purpose of our study was to compare the efficacy of CD47 mAb plus doxorubicin combination therapy in mouse models of osteosarcoma with CD47 mAb and doxorubicin monotherapy. Forty‐eight NOD scid gamma (NSG) mice with intratibial MNNG/HOS tumors received CD47 mAb, doxorubicin, combination therapy, or control IgG treatment. Twenty‐four mice (*n* = 6 per group) underwent pre‐ and post‐treatment magnetic resonance imaging (MRI) scans with the macrophage marker ferumoxytol, bioluminescence imaging, and histological analysis. Tumor ferumoxytol enhancement, tumor flux, and tumor‐associated macrophages (TAM) density were compared between different groups using a one‐way ANOVA. Twenty‐four additional NSG mice underwent survival analyses with Kaplan–Meier curves and a log‐rank (Mantel–Cox) test. Intratibial osteosarcomas demonstrated significantly stronger ferumoxytol enhancement and significantly increased TAM quantities after CD47 mAb plus doxorubicin combination therapy compared to CD47 mAb (*P* = 0.02) and doxorubicin monotherapy (*P* = 0.001). Tumor‐bearing mice treated with CD47 mAb plus doxorubicin combination therapy demonstrated significantly reduced tumor size and prolonged survival compared to control groups that received CD47 mAb (*P* = 0.03), doxorubicin monotherapy (*P* = 0.01), and control IgG (*P* = 0.001). In conclusion, CD47 mAb plus doxorubicin therapy demonstrates an additive therapeutic effect in mouse models of osteosarcomas, which can be monitored with an immediately clinically applicable MRI technique.

**Abbreviations**
BLI: bioluminescence imaging
MRI: magnetic resonance imaging
MSME: multislice multiecho
NSG: NOD.Cg‐*Prkdc* ^*scid*^ *Il2rg* ^*tm1Wjl*^/SzJ
TAM: tumor‐associated macrophages

## 1 Introduction

Therapy of high‐grade osteosarcomas includes chemotherapy with doxorubicin, cisplatin, and methotrexate and resection of the primary tumor (Bielack *et al*., 2002; Link *et al*., 1986; Marina *et al*., 2016). Unfortunately, the outcome of patients with metastatic disease remains dismal, with survival rates of 15–30% (Aljubran *et al*., 2009; Marina *et al*., 2016). Therefore, new therapy options for patients with metastasized osteosarcomas are urgently needed (Aljubran *et al*., 2009).

Tumor‐associated macrophages (TAMs) have been recognized as a new target for cancer immunotherapies (Cassetta and Kitamura, 2018). TAMs in high‐grade osteosarcoma are associated with reduced metastasis and improved survival (Buddingh *et al*., 2011). Recent clinical therapies have employed the use of TAM to directly attack tumor cells (Sikic *et al*., 2019; Takimoto *et al*., 2019; Yang and Zhang, 2017). Many osteosarcoma cells overexpress the surface marker CD47, which acts as a ‘don't eat me signal’ for TAM. Recent work in animal models has demonstrated that anticancer activity from TAM can be activated in sarcomas by blocking CD47 (Xu *et al*., 2015). Treatment with monoclonal antibodies against CD47 resulted in sarcoma cell phagocytosis by TAM and significant tumor growth reduction in mouse models (Edris *et al*., 2012; Herrmann *et al*., 2012). However, CD47 mAb monotherapy could not cure sarcoma‐bearing mice (Mohanty *et al*., 2019). As with other systemic therapies, CD47 mAb therapy will have to be combined with other therapies to maximize its efficacy.

**Figure 1:**
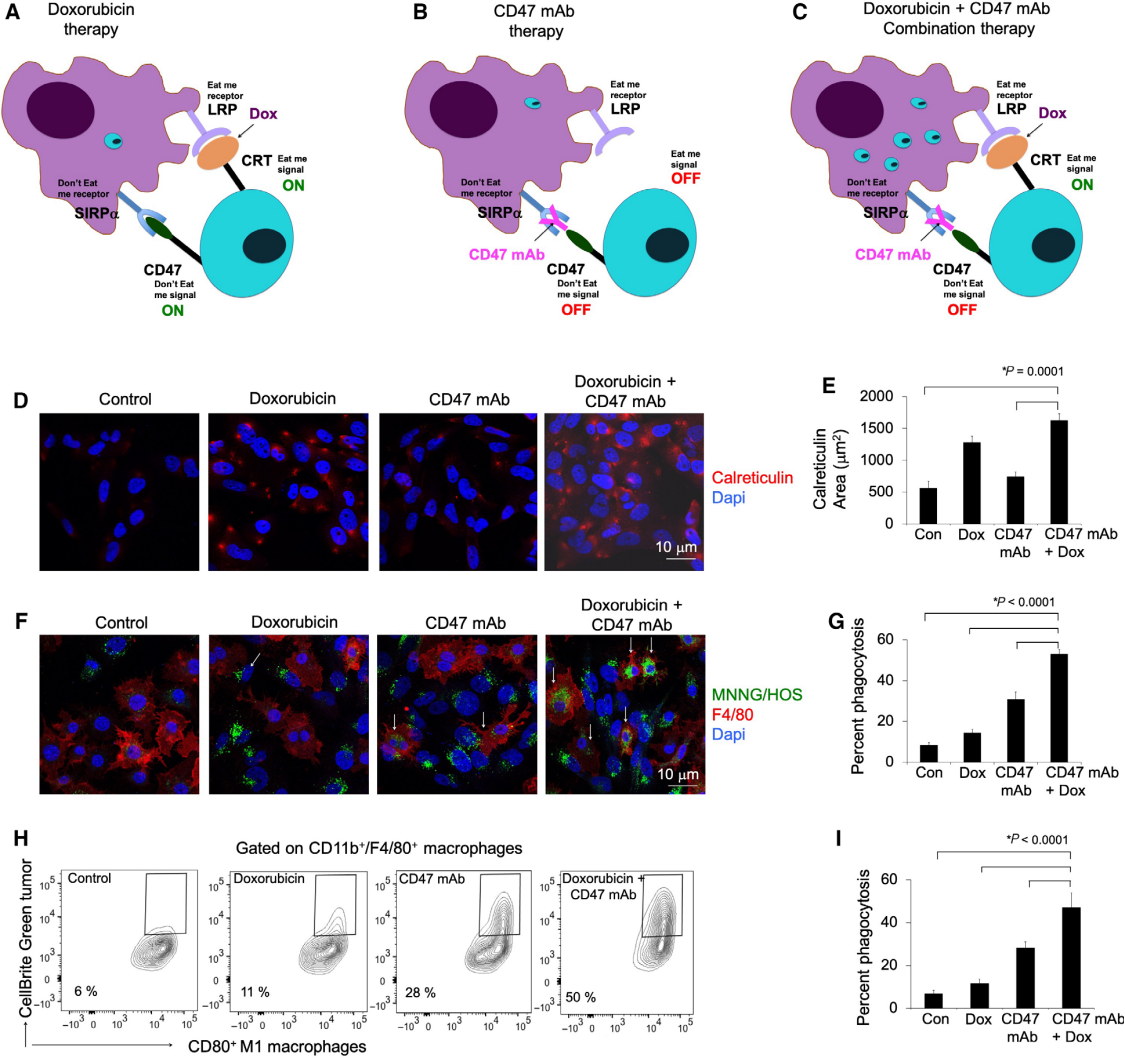
Doxorubicin enhances the phagocytic efficacy of CD47 mAb in osteosarcomas. Schematic demonstration of macrophage‐mediated tumor phagocytosis: (A) Doxorubicin therapy: Doxorubicin induces calreticulin (CRT) on the surface of tumor cells, which ‘turns on’ an eat‐me signal and enables binding to an eat‐me receptor on macrophages. However, CD47 expression on tumor cells counteracts calreticulin‐mediated phagocytosis, (B) CD47 mAb therapy: CD47 mAb block the interaction of tumor CD47 with SIRPα and ‘turns off’ the don't eat‐me signal, (C) combined doxorubicin and CD47 mAb combination therapy synergized by turning the eat‐me signal ‘on’ and don't eat‐me signal ‘off’, and inducing macrophage‐mediated tumor cell phagocytosis. (D) Representative calreticulin staining of MNNG/HOS tumor cells treated with IgG, doxorubicin (0.5 μm), CD47 mAb (10 μg·mL^−1^), and combination therapy. (E) Corresponding quantitative area of calreticulin staining of control and treated tumor cells. (F) For phagocytosis assays, MNNG/HOS tumor cells were cocultured with murine bone marrow‐derived M1 macrophages for 6 h. Confocal images of CellBrite green‐labeled MNNG/HOS tumor cells and F4/80^+^ macrophages in the presence of different therapeutics. Cells exposed to combination therapy show an increased quantity of phagocytized tumor cells in macrophages (arrows; scale bar 10 μm) compared to monotherapy. (G) Corresponding relative phagocytosis, calculated as the number of macrophages with phagocytized cancer cell divided by total macrophages per five high‐power field × 100%. (H) Flow cytometry contour plots of M1 macrophages uptaking control IgG and doxorubicin plus CD47mAb‐treated MNNG/HOS tumor cells and (I) corresponding charts showing tumor cell phagocytosis in control and treated sets. Data are displayed as means ± SD of *n* = 5 experiments per group, *P* value as indicated, one‐way ANOVA.

Doxorubicin is established for the treatment of osteosarcomas and acts on a common mechanistic pathway with CD47 mAb by inducing immunogenic cell death (Apetoh *et al*., 2007; Casares *et al*., 2005). Doxorubicin induces the expression of calreticulin on the cell surface of sarcoma cells that binds to low‐density lipoprotein receptor‐related protein 1 and functions as a prophagocytic ‘eat me’ signal for TAM (Chao *et al*., 2010; Fucikova *et al*., 2011). Previous studies have shown mixed findings for combination effects of anthracyclines with CD47 mAb. Suppression of CD47 chemosensitized hepatocellular carcinoma through blockade of CTSS/PAR2 signaling (Lee *et al*., 2014; Lo *et al*., 2016). In a different study, it has been shown that targeting CD47 enhanced the effect of doxorubicin chemotherapy *in vivo* by reducing tumor growth and metastatic spread by activation of an antitumor innate immune response (Feliz‐Mosquea *et al*., 2018; Iribarren *et al*., 2019). In contrast, Hermann *et al*. showed that CD7 mAb treatment did not further enhance macrophage‐mediated phagocytosis of anthracycline pretreated rhabdomyosarcoma cells (Herrmann *et al*., 2012). Anthracyclines have also been reported to downregulate CD47 levels in pancreatic cancer (Liu *et al*., 2017). It has been shown that in presence of CD47 binding peptide, camptothecin and doxorubicin‐induced proapoptotic activity was considerably inhibited (Rath *et al*., 2006). To our knowledge, antitumor effects of doxorubicin and CD47 mAb combination therapy have not been explored in osteosarcoma. We hypothesize that combined therapy of osteosarcomas with doxorubicin and CD47 mAb will significantly increase the efficacy of either drug alone (Fig. 1A–C).

To evaluate the effect of doxorubicin and CD47 mAb on TAM in osteosarcomas, we can leverage a new imaging technique that can noninvasively monitor the phagocytic activity of TAM *in vivo*. The approach relies on the FDA‐approved iron oxide nanoparticle compound ferumoxytol, which is phagocytosed by TAM and can be detected with clinical standard magnetic resonance imaging (MRI) technology. We previously showed that ferumoxytol‐MRI could detect TAM in osteosarcomas in mouse models (Mohanty *et al*., 2019) and in patients (Aghighi *et al*., 2018). We also showed that ferumoxytol‐MRI can monitor TAM response to immune‐modulating cancer therapies (Daldrup‐Link *et al*., 2011).

Thus, the purpose of our study was to compare the efficacy of CD47 mAb plus doxorubicin combination therapy in mouse models of osteosarcoma with CD47 mAb and doxorubicin monotherapy.

## 2 Materials and Methods

### 2.1 *In vitro* studies

MNNG/HOS cells (ATCC, Manassas, VA, USA) were grown in EMEM (ATCC, Manassas, VA, USA) supplemented with 10% FBS, 100 units·mL^−1^ of penicillin, and 100 mg·mL^−1^ of streptomycin. Cell lines used were authentic and confirmed to be mycoplasma negative using the MycoAlert Mycoplasma Activity kit (Lonza, Slough, UK). MNNG/HOS cells were engineered with luciferase‐td Tomato lentivirus. Lentiviral production and concentration were performed according to standard procedure. Osteosarcoma cells were transduced for 12 h at 37 °C, 5% CO_2_, with lentivirus containing 4 μg·mL^−1^ polybrene (Nitta *et al*., 2015). After 12 h, cells were washed repeatedly to remove extracellular lentivirus. Td Tomato‐positive osteosarcoma cells were sorted using a BD FACS ARIA (Becton Dickinson, Franklin Lakes, NJ, USA).

Calreticulin protein level in control and treated cells was evaluated by immunofluorescence staining as described previously (Mohanty *et al*., 2019; Zanganeh *et al*., 2016).

To evaluate macrophage‐mediated tumor phagocytosis (Mohanty *et al*., 2019; Zhang *et al*., 2016), in the presence of doxorubicin plus CD47 mAb combination therapy, MNNG/HOS osteosarcoma cells were labeled with 1,1′‐dioctadecyl‐3,3,3′,3′‐tetramethylindodicarbocyanine (CellBrite™ Green; Biotium, Fremont, CA, USA) according to the manufacturer's protocol and incubated at a 1 : 1 ratio with bone marrow‐derived M1 mouse macrophages in serum‐free IMDM, with 10 μg·mL^−1^ CD47 mAb, 500 nm doxorubicin, or both at 37 °C for 6 h. M1 polarization of macrophages was performed with previously established protocol (Mohanty *et al*., 2019). F4/80‐stained macrophages were assayed using Leica SP8 confocal microscopy. Tumor phagocytosis was calculated as the percentage of macrophages positive for phagocytized CellBrite™ Green^+^ cells. Tumor phagocytosis was also confirmed with flow cytometry on a BD FACS ARIA II flow cytometer. Fluorescently labeled antibodies targeting macrophage markers (CD11b, F4/80 and CD80) were used to identify the M1 population (11). Phagocytosis was quantified by the percentage of CellBrite™ Green events among CD11b^+^F4/80^+^CD80^+^ macrophage events.

### 2.2 *In vivo* studies

**Figure 2:**
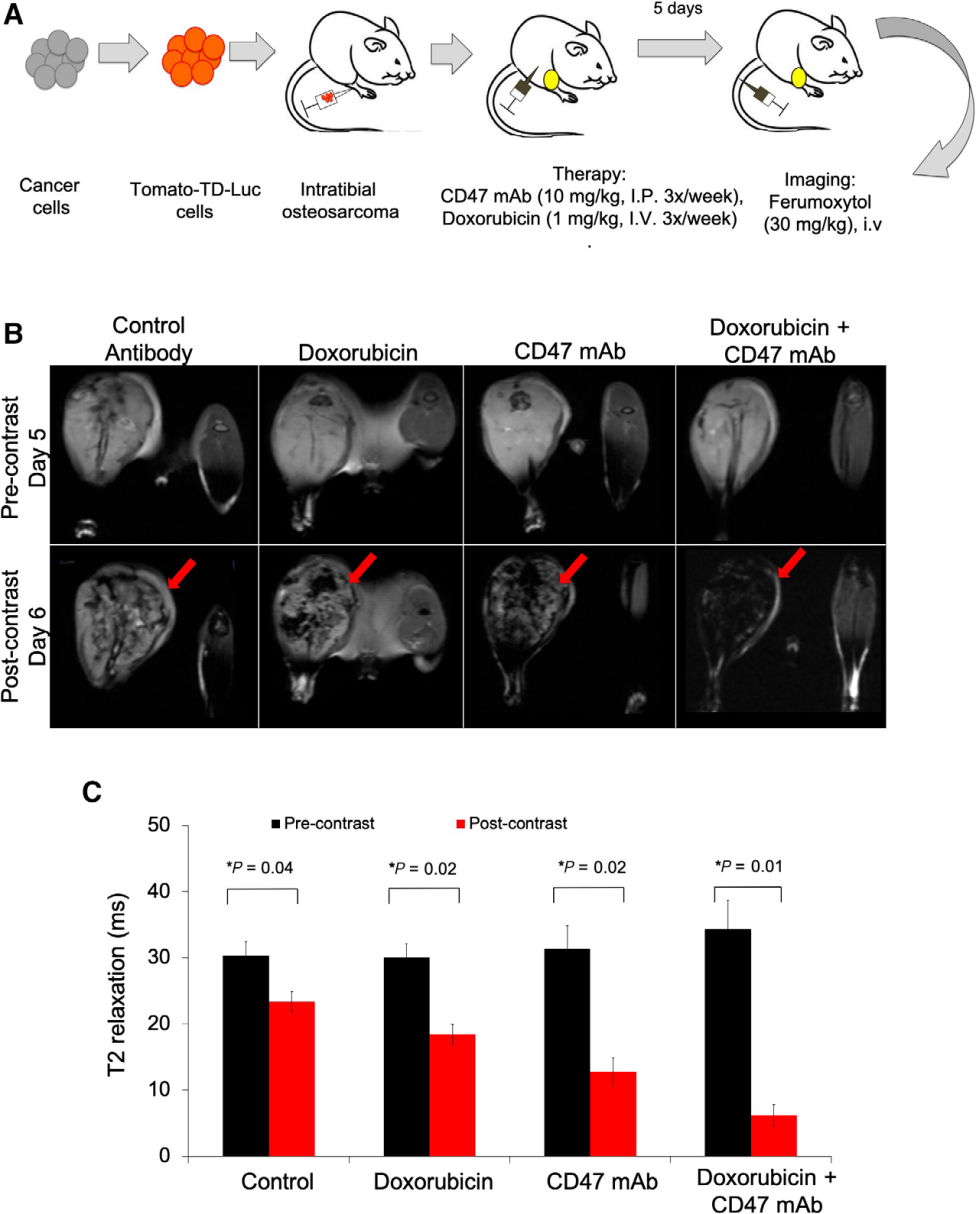
Ferumoxytol‐MRI shows increased T2 contrast in osteosarcomas after CD47 mAb combination therapy compared to monotherapy. (A) Schematic representation of experimental design: MNNG/HOS osteosarcoma cells were transfected with Tomato‐Td‐luciferase construct and implanted into the tibia of NSG mice (*n* = 6/group). Tumor‐bearing mice were treated with CD47 mAb (10 mg·kg^−1^, 3× per week) or doxorubicin (1 mg·kg^−1^, 3× per week) or combination therapy. Five days after therapy, MRI was performed prior to and 24‐h post‐ferumoxytol administration (i.v.). (B) Representative T2‐weighted MR images of MNNG/HOS tumors before (upper row) and at 24 h after (lower row) intravenous injection of the macrophage marker ferumoxytol. Ferumoxytol enhancement is demonstrated by dark (negative) tumor enhancement on T2‐weighted MR images (red arrows). (C) T2 relaxation times of control and treated tumors. T2 relaxation times (quantitative measures of dark tumor ferumoxytol enhancement) were measured on T2 maps, which were generated based on multiecho T2 MSME sequences. All results are represented as mean ± SD from six tumors per experimental group, *P*‐value as indicated, one‐way ANOVA.

Animal studies were approved by the Administrative Panel on Laboratory Animal Care at Stanford University (Protocol 24965). To establish orthotopic osteosarcomas, 48 NOD scid gamma (NSG) mice were injected intratibially with luciferase‐td Tomato expressing MNNG/HOS (5 × 10^5^) suspended in 20 μL PBS. Tibias of anesthetized mice were cleaned and injected with 20 μL of tumor cell suspension using a 27G tuberculin syringe (Fig. 2A). Tumor growth was confirmed with bioluminescence imaging (BLI). Twenty‐four xenografted mice were randomly divided into four groups (*n *=* *6 mice/group): Group 1 received a combination therapy of intravenous doxorubicin (Sigma, St. Louis, MO, USA) via the tail vein at a dose of 1 mg·kg^−1^ on days 0, 2, and 4 (3× per week, low‐dose schedule for minimal side effects) and intraperitoneal injections of CD47 mAb (clone B6H12; BioXcell, Lebanon, NH, USA) at a dose of 10 mg·kg^−1^ on days 1, 3, and 5 (3× per week). These doses were chosen based on the previous studies (Mohanty *et al*., 2019; Ren *et al*., 2008; Wang *et al*., 2010; Zhang *et al*., 2016). Group 2 received doxorubicin only and Group 3 received CD47 mAb only, using the same protocols as described above. Group 4 received intraperitoneal injections of control IgG (MOPC‐21; BioXcell) on days 1, 3, and 5 (3× per week).

Twenty‐four additional NOD scid gamma (NSG) mice with MNNG/HOS intratibial tumors were treated with doxorubicin plus CD47 mAb, doxorubicin only, CD47 mAb only or IgG, using the same protocols as above (*n* = 6 per group). Animal survival was evaluated from the first day of treatment until death. Body weight was measured twice a week. Animals were euthanized when turning moribund according to the above‐mentioned predefined criteria rapid weight loss, loss of ability to ambulate, labored respiration, or inability to drink or feed to avoid animal suffering.

### 2.3 MR imaging

After completion of control IgG, doxorubicin, CD47 mAb, CD47 mAb + doxorubicin therapy, all mice underwent MRI on a 7T MR scanner (Bruker‐Agilent Technologies‐General Electric Healthcare, Billerica, MA, USA) before intravenous injection of ferumoxytol and at 24 h after injection of ferumoxytol via the tail vein (Feraheme™; AMAG Pharmaceuticals, Waltham, MA, USA, 30 mg·kg^−1^). The following pulse sequences were used: T2‐weighted fast spin echo sequences with a repetition time (TR) of 4500 ms, an echo time (TE) of 42 ms, and a flip angle α: 90° and T2‐weighted multislice multiecho (MSME) sequences with a TR of 3000 ms, a TE of 8, 16, 24, 32, 40, 48, 56, 64, 72, 80, 88, and 96 ms and α: 90°. A field of view of 2 cm × 2 cm and a slice thickness of 0.5 mm for the MRI acquisitions. MSME images were used to create T2 maps and measure T2 relaxation times of the whole tumor with osirix software (Pixmeo, Geneva, Switzerland).

### 2.4 Bioluminescent imaging

Bioluminescent imaging was performed before therapy (day 0) as well as on day 5 (= directly after completion of therapy) and day 10 (= 5 days after completion of therapy), using an IVIS Spectrum scanner (Perkin Elmer Caliper Life Science, Waltham, MA, USA). D‐luciferin (firefly) potassium salt solution (Biosynth, Itasca, IL, USA; 15 mg·mL^−1^) was injected intraperitoneally (0.139 g luciferin per kg body weight), and mice were imaged until peak radiance was achieved. Total flux (photons per second) of osteosarcomas was measured by a single operator who was blinded to the experimental groups, using living image 4.0 software (Mohanty *et al*., 2017; Zanganeh *et al*., 2016). Total tumor flux (*y*‐axis) was plotted against different time points (*x*‐axis) to measure tumor growth overtime.

### 2.5 Immunocytochemistry

After completion of all imaging procedures, primary decalcified tumors and the bilateral lungs were explanted, fixed in 10% formalin, embedded in paraffin, cut at 5‐μm thickness, and processed for histology. Decalcification with 10% EDTA was performed as described previously (Belluoccio *et al*., 2013). For Prussian blue iron staining, tissues sections were deparaffinized with xylene, rehydrated, and stained according to the manufacturer's recommendation with the Sigma‐Aldrich Accustain Iron Stain Kit (Sigma‐Aldrich, St. Louis, MO, USA) (Mohanty *et al*., 2017). The DAB‐Quanto kit (Thermo Scientific, Waltham, MA, USA) was used to enhance Prussian blue stains. Sections were counterstained with nuclear fast red (Fisher Scientific, Hampton, NH, USA). For immunofluorescence, tumors were fixed in 4% paraformaldehyde and embedded in OCT embedding medium. Primary tumor sections were permeabilized with 0.1% Triton X‐100 in PBS for 10 min, washed 3 × 5 min in PBS, and blocked with 3% BSA in PBS for 30 min and stained for macrophages by incubation with F4/80 (1 : 100 dilution; R&D), CD80 (1 : 50 dilution; R&D, Minneapolis, MN, USA), iNOS (1 : 200 dilution; Abcam, Cambridge, MA, USA), calreticulin (1 : 200 dilution; Abcam) primary antibodies overnight at 4 °C before being washed 3× in PBS for 5 min each. Alexa Fluor 647‐conjugated secondary antibodies (1 : 200 dilution; Invitrogen) were added for 2 h in the dark and washed 3 × 5 min in PBS before samples were mounted with DAPI mounting media (Invitrogen, Carlsbard, CA, USA). Immunofluorescence images were acquired with a Leica SP8 confocal microscope using leica af software (Leica software, Leica Microsystems, Buffalo Groves, IL, USA) at 20× and 40× objectives. The percent area covered by Prussian Blue was quantitated using freely available imagej software (U. S. National Institutes of Health, Bethesda, MD, USA), using color thresholding followed by particle analysis (Aghighi *et al*., 2018). The percent area covered by F4/80^+^/CD80^+^/iNOS‐positive macrophages and calreticulin area in tumor xenografts was calculated by velocity software (Mohanty *et al*., 2019).

For histological analysis of lung metastases, coronal sections of the right and left lung were cut and stained with hematoxylin and eosin (H&E). Representative images were captured using an AxioImager Widefield Fluorescence Microscope (Zeiss, Thornwood, NY, USA) with a 20× objective for whole‐slide imaging. The summed area of metastases in the lungs (Krupnick *et al*., 2012) was measured with imagej software by one investigator, who was blinded to the treatment of the animals.

### 2.6 Statistical analysis

Tumor T2 relaxation time, BLI total flux measurements, the summed tumor area of metastases in the lungs, as well as the percent area of Prussian blue‐positive nanoparticles and F4/80^+^, CD80^+^, iNOS^+^ macrophages in primary tumors and calreticulin expression in tumors were compared between different experimental groups using exact one‐way analysis of variance (ANOVA). Kaplan–Meier survival curves were compared between different treatment groups using the log‐rank (Mantel–Cox) test. Statistical analyses were performed using graphpad prism (GraphPad, San Diego, CA, USA) software. The level of significance was set at *P *< 0.05 for all analyses.

## 3 Results

### 3.1 Combination treatment with doxorubicin and CD47 mAb enhances phagocytic elimination of osteosarcoma cells

Doxorubicin is known to be an inducer of immunogenic cell death (Apetoh *et al*., 2007) by triggering the surface expression of the tumor antigen calreticulin (Fucikova *et al*., 2011), which functions as an ‘eat me’ signal for TAM. To evaluate whether doxorubicin treatment induces calreticulin expression on MNNG/HOS tumor cells, we applied a calreticulin stain. We found increased calreticulin staining on the cell surface of MNNG/HOS tumor cells undergoing doxorubicin or combination treatment compared to PBS‐treated or CD47 mAb‐treated tumor cells (Fig. 1D,E, *P* = 0.0001).

To evaluate, whether calreticulin expression increases the phagocytic efficacy of CD47 mAb therapy, we cocultured MNNG/HOS tumor cells with bone marrow‐derived M1 macrophages in presence of doxorubicin, CD47 mAb, and combination treatment. We observed that tumor cells receiving combination treatment showed marked increase of phagocytosis compared with doxorubicin (3.6‐fold, *P* < 0.0001) or CD47 mAb treatments (1.7‐fold, *P* < 0.0001) both by confocal microscopy (Fig. 1F,G) and by FACS (Fig. 1H,I and Fig. S1).

### 3.2 Osteosarcomas treated with doxorubicin in combination with CD47 mAb showed increased ferumoxytol enhancement compared to monotherapy

Ferumoxytol serves as a TAM biomarker, which can be detected with MRI. To evaluate the *in vivo* TAM response to different immune‐modulating therapies, we performed ferumoxytol‐enhanced MRIs at day 6 after doxorubicin plus CD47 mAb combination therapy as well as doxorubicin, CD47 mAb and IgG monotherapy. In all treatment groups, precontrast MR images demonstrated bright (hyperintense) T2 signal of the primary tumor compared to skeletal muscle (Fig. 2B). At 24 h after injection of ferumoxytol, MR images demonstrated variable hypointense (dark) T2‐enhancement of the tumor tissue (Fig. 2B): Compared to precontrast images, quantitative T2 relaxation times were significantly decreased by 1.3‐fold after IgG treatment (*P* = 0.04), 1.6‐fold after doxorubicin treatment (*P* = 0.02), 2‐fold after CD47 treatment (*P* = 0.02), and 5.3‐fold decreased after doxorubicin plus CD47 mAb combination therapy (*P* = 0.01), indicating increasing nanoparticle retention in TAM (Fig. 2B,C).

### 3.3 Osteosarcomas treated with combination therapy contained increased quantities of iron nanoparticles and macrophages compared to monotherapy

**Figure 3:**
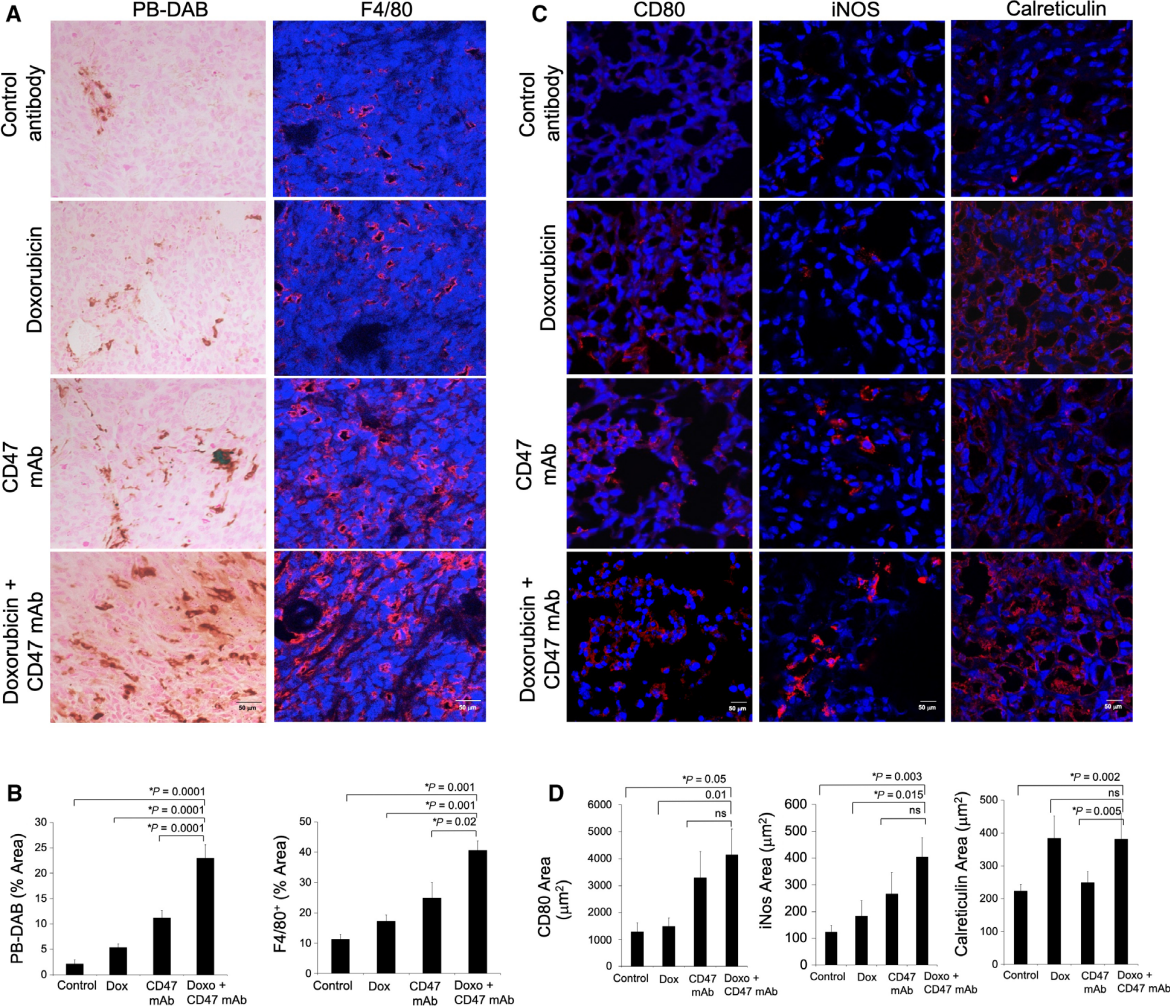
Histopathology shows M1 macrophage activation after doxorubicin and CD47 mAb therapy. (A) Representative Prussian blue‐DAB (scale bar 50 μm) iron stains and immunofluorescent F4/80 confocal images (scale bar 50 μm) of MNNG/HOS tumors show increasing quantities of iron oxide nanoparticles and macrophages in tumors treated with control Ab, CD47 mAb, doxorubicin, and combination therapies. (B) Corresponding quantitative area of Prussian blue‐DAB and F4/80‐positive macrophages in control and treated tumors. (C) Confocal immunofluorescent images and (D) Corresponding quantitative area of CD80, iNOS, and calreticulin staining in control and treated tumors (scale bar 50 μm). All results are represented as mean ± SD from six tumors per experimental group, *P*‐value as indicated, one‐way ANOVA.

To determine, whether the increased ferumoxytol‐MRI enhancement in osteosarcomas after combination therapy corresponded to increased quantities of activated macrophages in the tumor tissue, we performed immunohistochemistry stains against the iron oxide nanoparticles and TAMs. We found significantly larger summed areas of DAB‐Prussian blue iron staining in tumors treated with doxorubicin plus CD47 mAb combination therapy as compared to tumors treated with doxorubicin alone, CD47 mAb alone or control IgG alone (Fig. 3A,B, *P* = 0.0001). Immunofluorescence staining further showed increased quantities of F4/80^+^ macrophages in tumors treated with combination therapy when compared to doxorubicin alone (*P* = 0.001), CD47 mAb alone (*P* = 0.02), or control IgG alone (*P* = 0.001; Fig. 3A,B). We also performed immunofluorescence staining for M1 TAM activation markers‐CD80 and iNos and eat‐me receptor calreticulin on tumor cells. We observed that tumors receiving combination therapy demonstrated marked increase of CD80^+^ (*P* = 0.05, 0.01, ns) and iNos^+^ TAMs (*P* = 0.003, *P* = 0.015, ns) when compared to control, doxorubicin, or CD47 mAb‐treated tumors, respectively (Fig. 3C,D). We also observed increased staining intensity for calreticulin in tumors receiving combination therapy compared to control and CD47 mAb‐treated tumors (Fig. 3C,D, *P* = 0.002).

### 3.4 Doxorubicin plus CD47 mAb combination therapy significantly inhibited the growth of intratibial osteosarcomas and pulmonary metastases

**Figure 4:**
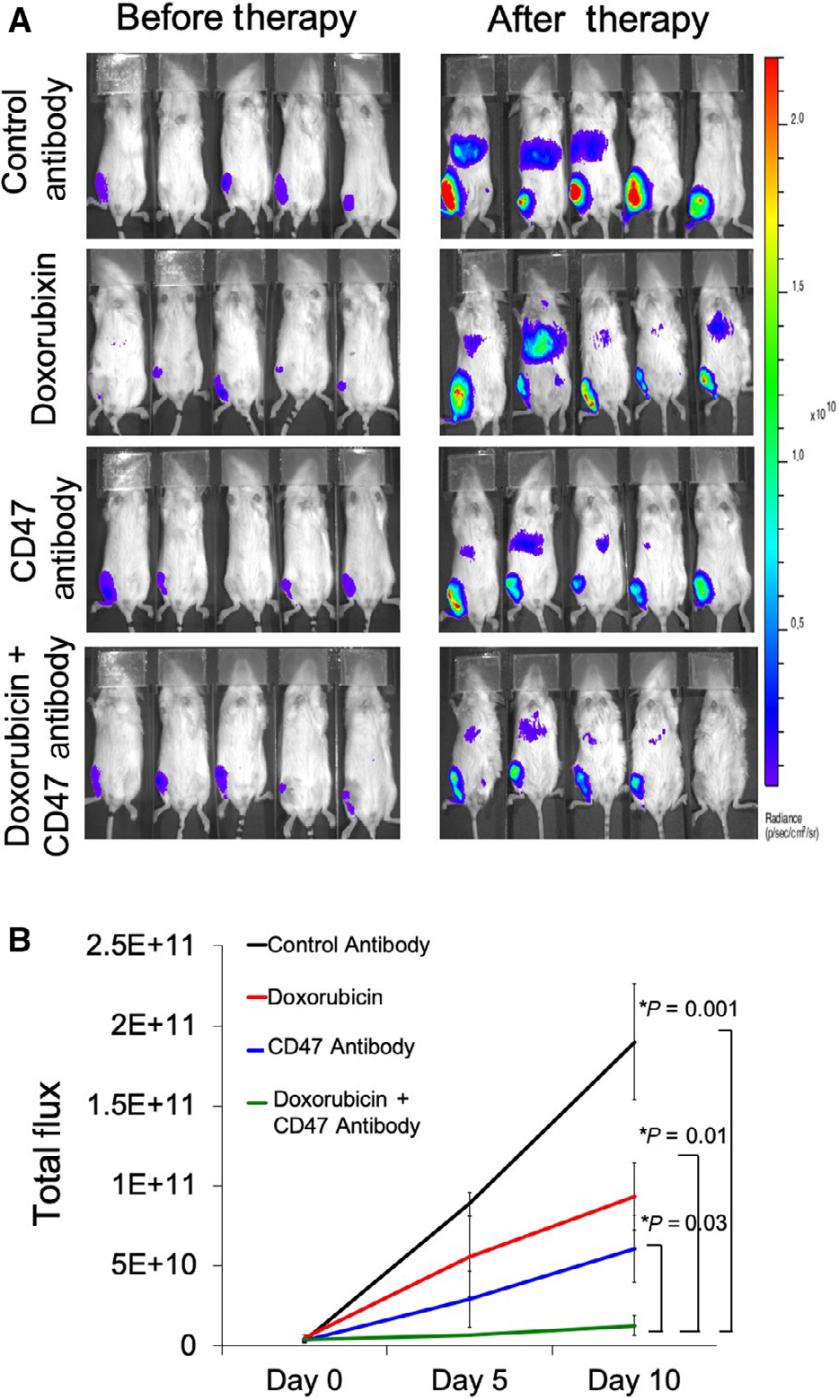
Bioluminescence imaging shows decreased tumor growth of osteosarcomas after doxorubicin and CD47 mAb combination therapy. (A) Bioluminescent *in vivo* images of mice with intratibial MNNG/HOS osteosarcomas before and after therapy with IgG, CD47 mAb, doxorubicin, and combination therapy. (B) Total quantified flux of MNNG/HOS osteosarcomas at different time points after intravenous treatment with IgG, doxorubicin, CD47 mAb, or combination therapy. Results are represented as mean ± SD from six tumors per experimental group, *P*‐value as indicated, one‐way ANOVA.

Bioluminescence imaging data showed a significantly decreased tumor burden in mice treated with doxorubicin plus CD47 mAb as compared to mice treated with doxorubicin only (*P* = 0.01), CD47 mAb only (*P* = 0.03), or control IgG (*P* = 0.001, Fig. 4A,B). Combining CD47 mAb with doxorubicin reduced the tumor flux 7‐fold compared with doxorubicin only and fourfold compared with CD47 mAb therapy only.

**Figure 5:**
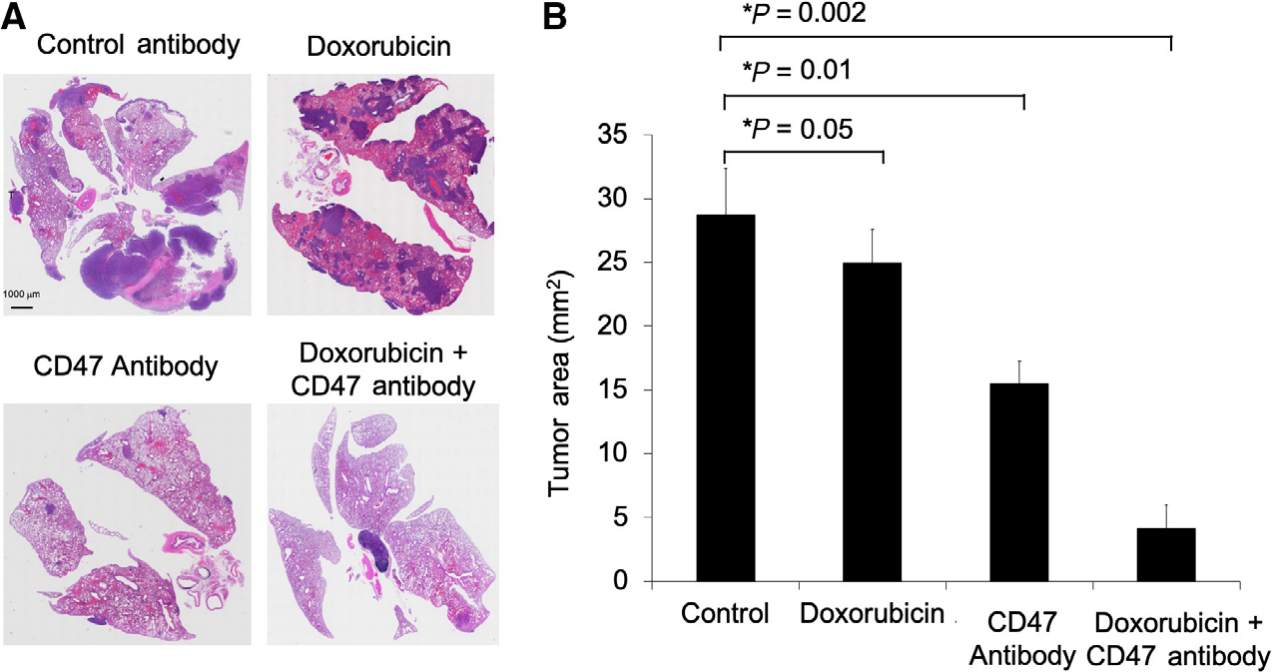
Doxorubicin plus CD47 mAb combination therapy prevents pulmonary metastasis in osteosarcoma‐bearing mice. (A) Low‐power (10×, H&E stain) view of the lungs showed metastasis from the primary tumor. (B) Corresponding summed tumor area of pulmonary metastases in mice treated with control IgG, doxorubicin, CD47 mAb, and combination therapy. Results are represented as mean ± SD from six animals per experimental group, *P*‐value as indicated, one‐way ANOVA.

Since MNNG/HOS intratibial tumors spontaneously metastasize to the lungs (Ren *et al*., 2015; Wan *et al*., 2009), we compared the tumor burden of lung metastasis in mice treated with doxorubicin plus CD47 mAbs to that of control groups. Lung sections from control IgG‐treated animals showed signs of metastasis from primary tumors (Fig. 5A,B). Compared to IgG‐treated controls, the summed tumor area on histopathological lung sections was not significantly different in doxorubicin‐treated mice (*P* = 0.05). However, the summed tumor area was 1.8‐fold reduced in CD47 mAb‐treated mice (*P* = 0.01) and 5‐fold reduced in doxorubicin plus CD47 mAb‐treated mice (*P* = 0.002) (Fig. 5A,B).

### 3.5 Combination therapy significantly improved survival of osteosarcoma‐bearing mice compared to monotherapy

**Figure 6:**
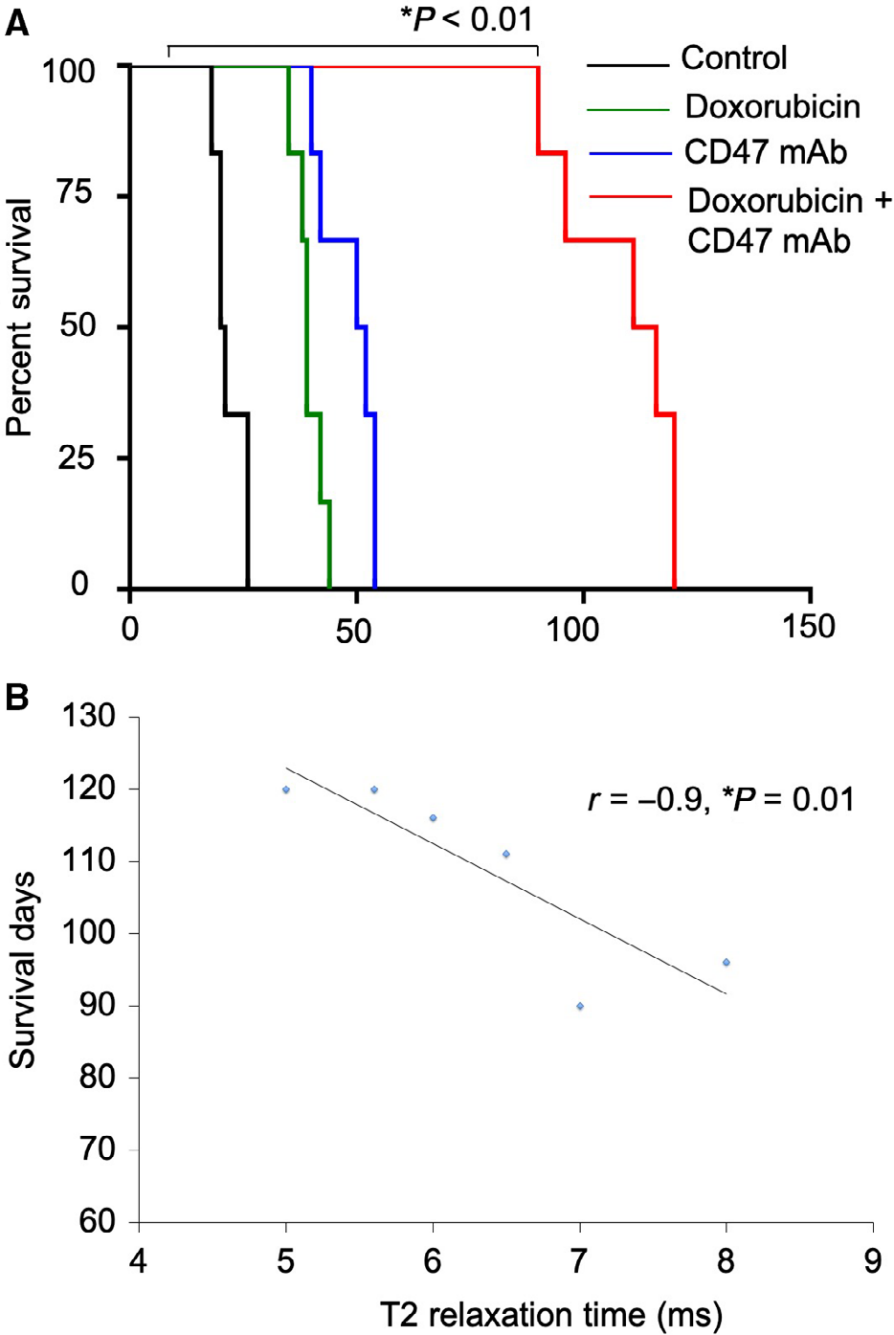
Doxorubicin plus CD47 mAb combination therapy improves survival in osteosarcoma‐bearing mice. (A) Kaplan–Meier survival curves demonstrate a significant survival benefit of combination therapy as compared to control, doxorubicin, and anti‐CD47 alone, log‐rank Mantel–Cox test (log‐rank Mantel–Cox test). (B) Tumor T2 relaxation times, obtained from ferumoxytol‐MRI, correlate with survival outcomes of mice receiving combination therapy (*r* = −0.9, *P* = 0.01, Spearman rank correlation, *n* = 6).

Kaplan–Meier survival curves showed that animals treated with doxorubicin plus CD47 mAb had significantly prolonged survival compared to animals treated with monotherapy (Fig. 6A). Compared to untreated controls, doxorubicin or CD47 mAb monotherapy survival was prolonged significantly in doxorubicin plus CD47 mAb‐treated mice (*P* = 0.007, Fig. 6A). In addition, ferumoxytol‐MRI could predict favorable outcomes. The degree of primary tumor ferumoxytol‐MRI enhancement at the end of combination therapy correlated with overall survival (*r* = −0.9, *P* = 0.01, Spearman rank correlation, Fig. 6B). These results suggest that ferumoxytol tumor enhancement can be used as a predictive biomarker for tumor response to doxorubicin and CD47 mAb therapy.

## 4 Discussion

Our data showed a strong additive effect of doxorubicin plus CD47 mAb combination therapy in mouse models of osteosarcomas, which led to significantly inhibited tumor growth and significantly improved survival of tumor‐bearing mice compared to CD47 mAb alone and doxorubicin alone. We postulate that macrophage‐mediated phagocytosis of tumor cells in the presence of CD47 mAb supports macrophage‐activating effects of doxorubicin. The NOD scid gamma (NSG) mouse strain is B‐ and T‐cell deficient, but has an intact macrophage response (Hu *et al*., 2011). This model allowed us to study macrophage responses to human xenografts, although it has to be recognized that results of CD47 mAb and doxorubicin combination therapies might be different in fully immunocompetent subjects. Several investigators, including Xu *et al*. (2015), and our own team (Mohanty *et al*., 2019) previously reported overexpression of CD47 on osteosarcoma cells and efficacy of CD47 mAb against human osteosarcomas in mouse models. Our team introduced ferumoxytol‐enhanced MRI as a new imaging biomarker for CD47 mAb‐mediated changes in TAM quantities and phagocytic activity in mouse models of MNNG/HOS osteosarcomas. We found that CD47 mAb triggered macrophage phagocytosis and tumoricidal effects in osteosarcomas and reduced tumor burden *in vivo*. We also found that the majority of cancer cells were phagocytosed alive and subsequently died in macrophages, while a smaller number of tumor cells died first and were secondarily phagocytosed (Mohanty *et al*., 2019). Studies by Xu *et al*. show that blockade of CD47 by specific Abs suppresses the invasive ability of osteosarcoma tumor cells and further inhibits spontaneous pulmonary metastasis of KRIB osteosarcoma cells *in vivo*. Although CD47mAb inhibited osteosarcoma growth in the above studies, combination therapy with antitumor chemotherapies is warranted to improve survival and reduce metastasis (Xu *et al*., 2015).

Combination therapy of doxorubicin and CD47 mAb has shown success in different cancers (Feliz‐Mosquea *et al*., 2018; Iribarren *et al*., 2019; Li *et al*., 2018; Wu *et al*., 2018) but their efficacy in osteosarcoma remains unknown. To our knowledge, our study is the first to evaluate the efficacy of combination therapy with doxorubicin in osteosarcomas. This is important, as monotherapies cannot reach complete tumor regression and future clinical translations will require integration of CD47 mAb therapies with classical chemotherapy.

Previous investigators studied other combinations of conventional cytotoxic drugs and immunotherapies to achieve enhanced effects and overcome tumor resistance to classical chemotherapy (Yan *et al*., 2018). Since cytotoxic drugs may be either immunostimulatory or immunosuppressive (Nowak *et al*., 2006), it is important to find synergistic combinations and recognize antagonistic combinations. In osteosarcomas, doxorubicin treatment increased the efficacy of immunotherapy with dendritic cells (Kawano *et al*., 2016) through similar mechanisms of HSP 70 and calreticulin activation, observed in our study. On the other hand, combining doxorubicin with PD‐L1 inhibitors showed a minimal survival advantage over PD‐L1‐monotherapy in K7M2 osteosarcoma model (Lussier *et al*., 2015). In this K7M2 metastatic osteosarcoma model, the authors show that α‐PD‐L1 mAb‐treated mice demonstrated an adaptive resistance mechanism in the microenvironment where the tumor or tumor microenvironment may be using CTLA‐4 ligation as an alternative pathway to escape immune destruction. Thus, combining doxorubicin chemotherapy with PD‐L1 blockade immunotherapy did not appear to have additional beneficial effects on tumor control. On the other hand, in patients with lymphoma, a recent phase 1b study showed that CD47 mAb in concert with rituximab showed enhanced effects by enhancing macrophage‐mediated antibody‐dependent cellular phagocytosis (Advani *et al*., 2018). The authors achieved 33% complete response in patients with diffuse large B‐cell lymphoma and 43% complete response in patients with follicular lymphoma (Advani *et al*., 2018). In a mouse model of breast cancer, Feliz‐Mosquea *et al*. (2018) found significantly reduced metastasis after doxorubicin and CD47 mAb combination therapy.

With the introduction of new TAM‐modulating immunotherapies to the clinic, it becomes increasingly important to identify and stratify responders and nonresponders to these therapies. Since tumor response to new immunotherapies does not lead to a decline in tumor size, at least not in the immediate post‐treatment phase, new tools for treatment monitoring are required. Various diagnostic tools for TAM detection and quantification have been developed, including gene expression analyses, immunohistochemistry, and fluorescent magnetic nanoparticle labeling (Almatroodi *et al*., 2016; Kung *et al*., 2017; Sikandar *et al*., 2017). However, a major disadvantage of these methods is their invasive nature and lack of clinical availability. We established ferumoxytol‐MRI as a new imaging biomarker for tumor response to TAM‐modulating therapies in mouse models of breast cancer (Daldrup‐Link *et al*., 2011), brain cancer (Mohanty *et al*., 2017), and osteosarcomas (Mohanty *et al*., 2019). In addition, we recently showed that ferumoxytol‐MRI can quantify TAM quantities in patients with osteosarcoma and lymphoma (Aghighi *et al*., 2018) and brain cancer (Iv *et al*., 2018). A major side effect of anti‐CD47 treatment is anemia. Ferumoxytol is an FDA‐approved iron supplement for anemia treatment and might counteract this side effect (Daldrup‐Link *et al*., 2011; Muehe *et al*., 2015). Ferumoxytol can rarely lead to allergic and anaphylactic reactions. Fortunately, we have not encountered major side effects thus far in our patients with osteosarcomas (Muehe *et al*., 2015). We carefully screen our patients for any history of allergic reactions and exclude patients with risk of allergies from ferumoxytol exposure.

## 5 Conclusions

- CD47 mAb plus doxorubicin combination therapy improves survival in osteosarcoma‐bearing mice.
- Ferumoxytol‐MRI can monitor TAM responses of human osteosarcomas to CD47 mAb plus doxorubicin combination therapy *in vivo*, in a mouse model.
- The described new therapy approach and imaging tool for monitoring treatment response are immediately clinically translatable.

## Conflict of interest

The authors declare no conflict of interest.

## Author contributions

SM and HED‐L designed the experiments. SM, MA, KY, and JLT performed the *in vitro* and *in vivo* experiments. SM, MA, KY, and HED‐L analyzed the data and prepared the figures. SM and HED‐L wrote the manuscript. HED‐L reviewed the data and edited the manuscript.

## Supporting information


**Fig. S1.** Gating strategy for macrophage‐mediated tumor phagocytosis.
