# Supplementary figures and images for: Improving the efficacy of osteosarcoma therapy: combining drugs that turn cancer cell ‘don't eat me’ signals off and ‘eat me’ signals on
Source: Mol Oncol. 2019 Aug 13;13(10):2049–61. doi: 10.1002/1878-0261.12556 (PMC6763764; doi:10.1002/1878-0261.12556)

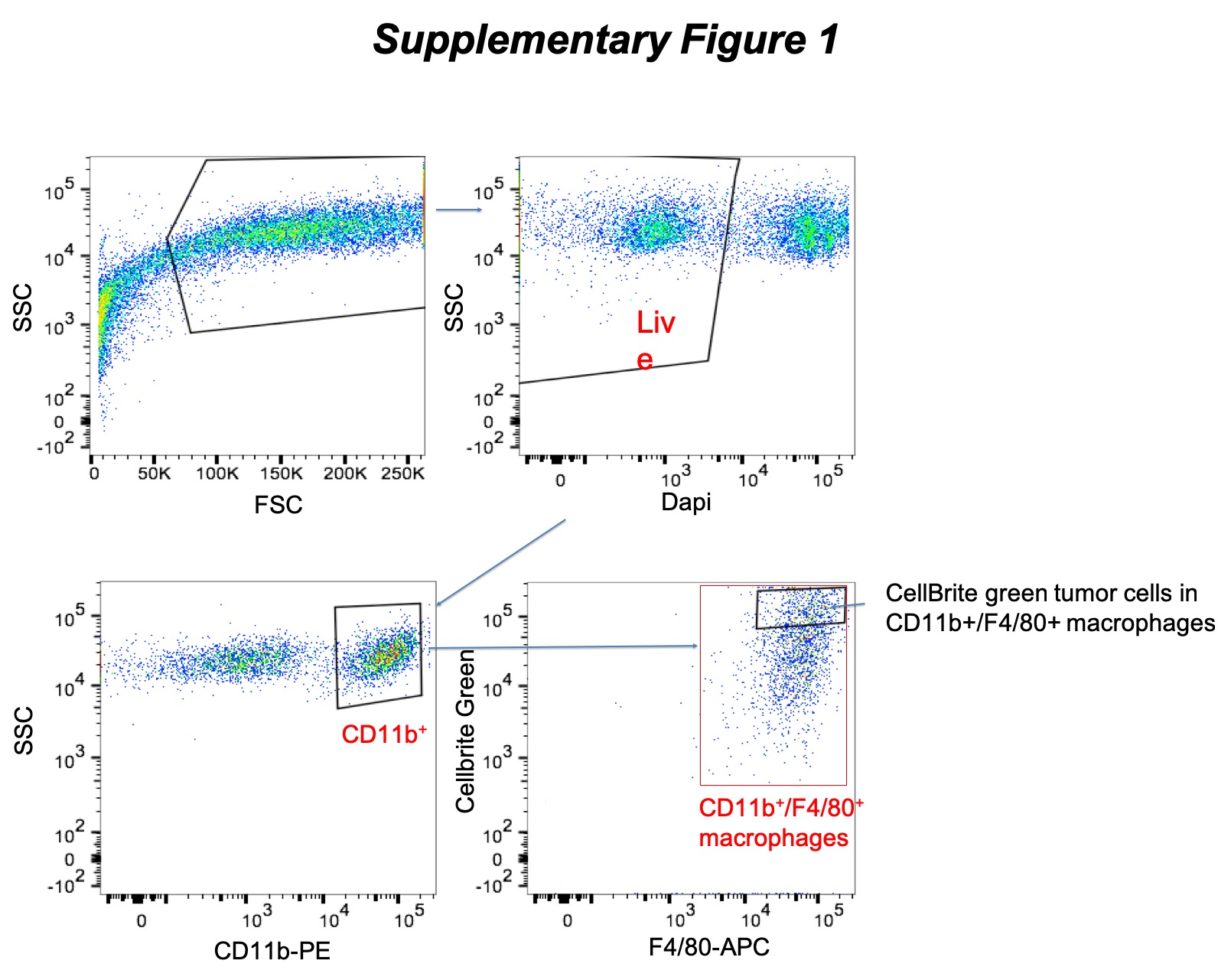

Supplement: Supplementary file 1 — Fig. S1. Gating strategy for macrophage‐mediated tumor phagocytosis. [file MOL2-13-2049-s001.jpg]
