# Supplementary material for: Improving the efficacy of osteosarcoma therapy: combining drugs that turn cancer cell ‘don't eat me’ signals off and ‘eat me’ signals on
Source: Mol Oncol. 2019 Aug 13;13(10):2049–61. doi: 10.1002/1878-0261.12556 (PMC6763764; doi:10.1002/1878-0261.12556)
Supplement: Supplementary file 2 [file MOL2-13-2049-s002.docx]

**Supplementary Information**

**Supplementary figure 1.** Representative dot plot describing gating strategy to determine tumor phagocytosis by murine macrophages in presence of control and anti-CD47 antibodies. Murine macrophages were co-cultured with cellbrite green-labeled tumor cells in presence of control and anti-CD47 antibodies. Macrophages were gated on Dapi^-^CD11b^+^F4/80^+^ populations. Phagocytosis was determined by percentage of cellbrite green cells in macrophage-gated population.
